# Psychosocial outcomes in adult men born with hypospadias: A register-based study

**DOI:** 10.1371/journal.pone.0174923

**Published:** 2017-04-06

**Authors:** Anna Skarin Nordenvall, Christina Norrby, Agnieszka Butwicka, Louise Frisén, Anna Nordenström, Catarina Almqvist, Agneta Nordenskjöld

**Affiliations:** 1 Department of Women's and Children's Health, and Center for Molecular Medicine, Karolinska Institutet, Stockholm, Sweden; 2 Department of Pediatric Surgery, Astrid Lindgren Children´s Hospital, Karolinska University Hospital, Stockholm, Sweden; 3 Department of Medical Epidemiology and Biostatistics, Karolinska Institutet, Stockholm, Sweden; 4 Department of Child Psychiatry, Medical University of Warsaw, Warsaw, Poland; 5 Department of Clinical Neuroscience, Karolinska Institutet, Stockholm, Sweden; 6 Child and Adolescent Psychiatry Research Center, Stockholm, Sweden; 7 Department of Women's and Children's Health, Karolinska Institutet, Stockholm, Sweden; 8 Department of Pediatric Endocrinology, Astrid Lindgren Children’s Hospital, Karolinska University Hospital, Stockholm, Sweden; 9 Lung and Allergy Unit, Astrid Lindgren Children's Hospital, Karolinska University Hospital, Stockholm, Sweden; Jichi Medical University, JAPAN

## Abstract

In this nationwide matched cohort study, we have investigated whether being born with hypospadias affect subsequent psychosocial outcomes in adulthood. We analyzed prospectively collected data from national Swedish registers. Data on the diagnoses were collected from the National Patient Register and the Medical Birth Register. Data on psychosocial outcomes such as educational and income level, marital status and disability pension were collected from Statistics Sweden. The effects of covariates, such as age, county of birth, presence of other malformations and psychiatric illness, were taken into account. The associations between hypospadias and psychosocial outcomes were calculated using conditional logistic regression and expressed as odds ratios (OR) and 95% confidence intervals (CI). We included 4378 men diagnosed with hypospadias, born between 1969 and 1993 in Sweden. Patients with hypospadias were matched with unaffected men by year of birth and birth county. We did not detect any differences in educational or income level. The probability of entering marriage (OR 1.02, 95% CI 0.90–1.14) did not differ, regardless of phenotype. We did, however, detect a 40% increased probability of receiving a disability pension, (OR 1.39, 95% CI 1.20–1.61). In conclusion, men born with hypospadias in Sweden do not differ from unaffected men with respect to the majority of psychosocial outcomes studied. They are, however, at increased risk of receiving a disability pension, which motivates further investigations.

## Introduction

When a child is born with a birth defect, it is a critical task for healthcare to provide answers to the questions raised by the worried parents. These questions often concern the reason for the malformation and how it may affect the child’s future. Questions regarding well-being, future education and family formation are frequently raised at an early stage.

Hypospadias is one of the most common congenital malformations affecting boys. It is usually diagnosed at birth and is characterized by misplacement of the urethral meatus ventrally and proximally from the tip of the glans penis. The phenotype ranges from distal cases with the meatus positioned on the ventral side of the glans, that can be regarded as a cosmetic problem, to successively more proximal cases with penoscrotal, scrotal or perineal meatus. The proximal cases may be associated with bifid scrotum, penile curvature, penoscrotal transposition and sometimes uncertain sex at birth.

The malformation is suggested to have both genetic and environmental causes[1]. Molecular diagnosis is rarely established, especially in the distal cases. Associations with clinical risk factors, such as low birth weight, maternal hypertension and pre-eclampsia have been demonstrated repeatedly[2]. Hypospadias may occur in association with one or several other malformations.

Untreated hypospadias may result in functional problems involving urination and intercourse, especially in the penile and more proximal cases[3]. In Sweden the malformation is most commonly treated surgically, usually by a single-step procedure, but, in severe cases, multiple surgical interventions may be required. Surgery is primarily performed at one to two years of age in Sweden. The surgical methods have evolved and improved throughout the years. Historically, hypospadias surgery required several days of hospitalization; nowadays, most patients undergo outpatient surgery or stay only overnight. Urethral fistulas and strictures are complications that may arise and lead to repeated surgical intervention[4].

Studies on the implications of being born with hypospadias have mainly focused on functional outcomes that strongly correlate with the surgery, such as cosmetics and sexual function. For example, males operated on for hypospadias are reported to be less satisfied with their urinary [5, 6] and sexual function, compared to controls.[7]

Previous studies have investigated the psychological impact of being born with hypospadias and have come to worrying results suggesting that boys born with the condition have more behavioral problems during childhood and adulthood[8] and that poor cosmetic results are associated with worse school performance.[9] Additionally, a few studies have shown an association between hypospadias and psychiatric disorders.[10, 11] However, other studies contradict these results by not identifying any differences in psychosocial and psychosexual adjustment[12] or socioeconomic status.[13, 14]

Nonetheless these previous studies raised our concerns that men with hypospadias might have impaired psychosocial outcomes as compared to non-affected men.

To date, no large-scale register-based studies have investigated educational and income levels, marital status or disability pensions in men born with hypospadias. In order to improve the information given to parents and identify areas where follow-up may be enhanced, we have investigated whether men born with hypospadias differ from unaffected men regarding psychosocial outcomes in adulthood.

## Material and methods

In Sweden, all citizens have a unique personal identity number that enables linkage between the national health registers. We performed a matched cohort study using prospectively collected data from registers covering healthcare held by the Swedish National Board of Health and Welfare and registers covering socioeconomic data held by Statistics Sweden. Specifically we used data from the Medical Birth Register (MBR; 1973 and onward), the Multi-Generation Register (MGR), the National Patient Register (NPR; established in 1960’s, national coverage since 1987, including outpatient visits since 2001), the Longitudinal Integration Database for Health Insurance and Labor Market Studies (Swedish acronym, LISA; 1990 and onward), the Population and Housing Census from 1975, -80 and -85 (Swedish acronym, FoB), the Register of Education (Swedish acronym, UREG; 1985 and onward) and the Grade 9 School Marks Registers (available from 1998 and onward).

### Study design and participants

From the NPR and MBR, we identified men born in Sweden between January 1, 1969, and December 31, 1993, with a registered diagnosis of hypospadias. The NPR started collecting information on all Swedish inpatient care at public hospitals in the 1960s, initially covering only a few of Sweden’s counties, but expanded step by step to national coverage as of 1987. As a result of the coverage by the NPR, we were able to assemble a cohort that gradually expanded county by county from 1969 to 1986, after which it became a national cohort. Each patient with hypospadias was matched with 100 unaffected males by birth county and birth year. Children who emigrated before the age of 16 were excluded and subjects were observed until December 31, 2009.

### Definition of exposure

Data on ascertainment of hypospadias were collected from the MBR and NPR. Hypospadias surgery was carried out at inpatient visits during the years from which the patients were included, making it unlikely that any patient with hypospadias who required surgery were not included. Three different ICD coding systems cover the study period and therefore different codes had to be used to identify patients (752.2 in ICD-8, 752.6 in ICD-9 and Q54 in ICD10). In the subanalysis more specified subgrouping where performed, glandular and penile hypospadias were both regarded as distal, since further subgrouping between the both was not possible until 1997 due to ICD-8 and ICD-9 coding standards. Penoscrotal and perineal hypospadias was possible to sub classify in both ICD-8 and -10. The men who had received this coding were however few, and had sometimes been registered as both phenotypes; the codes were therefore grouped and defined as proximal. If the boy had received more than one classification of hypospadias, the most severe classification was used in the analysis.

### Psychosocial outcomes

Data on the highest level of education achieved were collected from FOB, UREG and LISA and were categorized as compulsory school (≤9 years of education), high school (10–12 years) and college graduate or higher education (≥15 years). Information on eligibility for high school (passing grades in eight subjects, including Swedish, English and Mathematics) was collected for a subgroup of the cohort since this variable is available in the Grade 9 School Marks Registers only from 1998 and onward, accordingly only men born 1983 and onward were included in these analyses to assure they had attained an age of 15 years in 1998. Data on disposable income were obtained from FoB -75, -80 and -85; from 1990, annual disposable income was obtained from LISA. For each available year, the income levels in the total population were divided into quintiles. Thereafter, individuals were allocated to either a low (1st quintile), median (2nd–4th quintile) or high (5th quintile) income level. The highest level of income achieved was used as reference.

Information on whether the individual ever entered marriage was collected from FoB -80, -85 and LISA. If registered as divorced without information on marriage, the individual was categorized as being married at some point in time. Information on disability pension was obtained from LISA.

### Covariates

County of birth and birth year served as matching variables and were collected from Statistics Sweden. Other covariates used in regression analyses were the presence of any other malformation, excluding genital organs, collected from the MBR or NPR, and gestational age and weight at birth, collected from the MBR. Sensitivity analyses, stratified by the presence of a psychiatric disorder previously shown to be associated with hypospadias,[11] were performed, data were collected from the NPR and subcategorized into autism spectrum disorders (ASD), attention deficit hyperactivity disorder (ADHD), behavioral/emotional disorders (BEDs) and intellectual disability (ID).

### Statistics

The association between hypospadias and psychosocial outcomes was estimated using conditional logistic regression and expressed by odds ratios with 95% confidence intervals. In analyses regarding the highest levels of education and income, high school education and the median income served as references in the respective analyses. Marital status and ever having received a disability pension were analyzed as binary outcomes. Directed acyclic graphs and stratification were used to determine potential confounders for different models. Calculations were performed using SAS^®^ versions 9.3 and 9.4 (Statistical Analysis Systems). The Ethics Committee of Karolinska Institutet approved the study.

## Results

We included 4,378 men diagnosed with hypospadias and born between 1969 and 1993. Characteristics of the study population are outlined in Table 1. Fifty-eight percent of the population could be classified according to phenotype; 42% were either diagnosed as hypospadias not other specified (NOS) or diagnosed during the period when ICD-9 was used without the possibility to subclassify.

**Table 1:** Characteristics of the study population.

	Total study population		Distal hypospadias		Proximal hypospadias		Hypospadias with unclassified phenotype	
	cases	non-cases	cases	non-cases	cases	non-cases	cases	non-cases
	n (%)	n (%)	n (%)	n (%)	n (%)	n (%)	n (%)	n (%)
	4738	473800	2512 (53.0)	251200	217 (4.6)	21700	2009 (42.4)	200900
**Birth year**								
1969–1977	915 (19.3)	91500	741 (29.5)	74100	33 (15.2)	3300	141 (7.0)	14100
1978–1985	1423 (30.0)	142300	1053 (41.9)	105300	62 (28.6)	6200	308 (15.3)	30800
1986–1993	2400 (50.7)	240000	718 (28.6)	71800	122 (56.2)	12200	1560 (77.7)	156000
Eligible for upper secondary*								
No	342 (7.2)	29292 (6.2)	142 (5.7)	10611 (4.2)	7 (3.2)	1461 (6.7)	193 (9.6)	17220 (8.6)
Yes	2323 (49.0)	246830 (52.1)	852 (33.9)	89456 (35.6)	118 (54.4)	12725 (58.6)	1353 (67.3)	144649 (72.0)
Missing	2073 (43.8)	197678 (41.7)	1518 (60.4)	151133 (60.2)	92 (42.4)	7514 (34.6)	463 (23.0)	39031 (19.4)
**Highest level of education**								
Less than elementary school or no data	357 (7.5)	25254 (5.3)	146 (5.8)	12763 (5.1)	28 (12.9)	1211 (5.6)	183 (9.1)	11280 (5.6)
Elementary school	1413 (29.8)	140666 (29.7)	588 (23.4)	57576 (22.9)	70 (32.3)	7436 (34.3)	755 (37.6)	75654 (37.7)
Upper secondary school	1940 (40.9)	204665 (43.2)	1062 (42.3)	109016 (43.4)	80 (36.9)	8954 (41.3)	798 (39.7)	86695 (43.2)
College	1028 (21.7)	103215 (21.8)	716 (28.5)	71845 (28.6)	39 (18.0)	4099 (18.9)	273 (13.6)	27271 (13.6)
**Highest level of income**								
No income	89 (1.9)	7234 (1.5)	47 (1.9)	4107 (1.6)	9 (4.1)	357 (1.6)	33 (1.6)	2770 (1.4)
Low income	1700 (35.9)	171356 (36.2)	575 (22.9)	56556 (22.5)	92 (42.4)	9280 (42.8)	1033 (51.4)	105520 (52.5)
Median income	1928 (40.7)	189620 (40.0)	1167 (46.5)	112336 (44.7)	83 (38.2)	7913 (36.5)	678 (33.7)	69371 (34.5)
High income	876 (18.5)	92142 (19.4)	676 (26.9)	71574 (28.5)	29 (13.4)	3491 (16.1)	171 (8.5)	17077 (8.5)
Missing	145 (3.1)	13448 (2.8)	47 (1.9)	6627 (2.6)	4 (1.8)	659 (3.0)	94 (4.7)	6162 (3.1)
**Married**								
No	4260 (89.9)	426899 (90.1)	2135 (85.0)	214175 (85.3)	202 (93.1)	19955 (92.0)	1923 (95.7)	192769 (96.0)
Yes	478 (10.1)	46901 (9.9)	377 (15.0)	37025 (14.7)	15 (6.9)	1745 (8.0)	86 (4.3)	8131 (4.0)
**Disability pension**								
No	4344 (91.7)	445782 (94.1)	2328 (92.7)	235506 (93.8)	192 (88.5)	20393 (94.0)	1824 (90.8)	189883 (94.5)
Yes	221 (4.7)	11412 (2.4)	113 (4.5)	6662 (2.7)	19 (8.8)	501 (2.3)	89 (4.4)	4249 (2.1)
Missing	173 (3.7)	16606 (3.5)	71 (2.8)	9032 (3.6)	6 (2.8)	806 (3.7)	96 (4.8)	6768 (3.4)
**Additional malformations**	816 (17.2)	23186 (4.9)	396 (15.8)	11366 (4.5)	76 (35.0)	1112 (5.1)	344 (17.1)	10708 (5.3)
Preterm delivery, <37 weeks**								
Yes	534 (11.3)	27958 (5.9)	241 (9.6)	14289 (5.7)	46 (21.2)	1300 (6.0)	247 (12.3)	12369 (6.2)
No	3967 (83.7)	420676 (88.8)	2141 (85.2)	222138 (88.4)	161 (74.2)	19226 (88.6)	1665 (82.9)	179312 (89.3)
Missing	237 (5.0)	25166 (5.3)	130 (5.2)	14773 (5.9)	10 (4.6)	1174 (5.4)	97 (4.8)	9219 (4.6)
Birth weight**								
<2500g	497 (10.5)	18161 (3.8)	205 (8.2)	9396 (3.7)	58 (26.7)	893 (4.1)	234 (11.6)	7872 (3.9)
Missing	238 (5.0)	24860 (5.2)	130 (5.2)	14587 (5.8)	11 (5.1)	1181 (5.4)	97 (4.8)	9092 (4.5)
**Psychiatric diagnoses**								
ASD, ADHD, Beh or Int Dis	265 (5.6)	16097 (3.4)	119 (4.7)	7087 (2.8)	22 (10.1)	754 (3.5)	124 (6.2)	8256 (4.1)
Autism Spectrum Disorder	62 (1.3)	4007 (0.8)	25 (1.0)	1709 (0.7)	6 (2.8)	193 (0.9)	31 (1.5)	2105 (1.0)
ADHD	100 (2.1)	7122 (1.5)	42 (1.7)	2971 (1.2)	6 (2.8)	350 (1.6)	52 (2.6)	3801 (1.9)
Behavioral/emotional disorders	85 (1.8)	5828 (1.2)	38 (1.5)	2497 (1.0)	6 (2.8)	283 (1.3)	41 (2.0)	3048 (1.5)
Intellectual disability	108 (2.3)	3680 (0.8)	50 (2.0)	1750 (0.7)	13 (6.0)	172 (0.8)	45 (2.2)	1758 (0.9)

*Data available from 1998

** Data available from 1973

Few differences were observed between men with hypospadias and those without, apart from perinatal characteristics, such as the associated malformations and being born preterm. In addition, men with hypospadias were more often affected by psychiatric disease.

### All hypospadias cases

In the crude conditional logistic regression model, men with hypospadias, in the aggregate, were less likely to be eligible for upper secondary school and less likely to be in the high-income group (Table 2).

**Table 2:** Logistic regression models for the associations between hypospadias and socioeconomic outcomes, regardless of phenotype.

	Crude	Model 1 *	Model 2 **	Model 3 ***
	OR (95% CI)	OR (95% CI)	OR (95% CI)	OR (95% CI)
Entered marriage	1.03 (0.92–1.14)	1.05 (0.95–1.17)	0.99 (0.88–1.12)	1.02 (0.90–1.14)
Disability pension	2.01 (1.75–2.30)	1.46 (1.27–1.69)	1.87 (1.62–2.15)	1.39 (1.20–1.61)
Eligible for upper secondary†	0.81 (0.72–0.90)	0.82 (0.74–0.92)	0.86 (0.77–0.96)	0.87 (0.78–0.98)
**Highest level of education achieved**				
Elementary school	1.15 (1.04–1.27)	1.12 (1.01–1.24)	1.11 (1.00–1.23)	1.09 (0.99–1.20)
Upper secondary school	1	1	1	1
College	1.04 (0.96–1.13)	1.05 (0.97–1.14)	1.06 (0.98–1.16)	1.07 (0.98–1.17)
**Highest level of income**				
Low income	1.01 (0.91–1.13)	1.00 (0.90–1.11)	1.01 (0.90–1.12)	0.99 (0.89–1.10)
Median income	1	1	1	1
High income	0.90 (0.82–0.99)	0.93 (0.84–1.01)	0.93 (0.84–1.02)	0.95 (0.86–1.04)

^†^ Men born 1983 and onward included

* Other malformations

** Gestational age and birth weight

*** Other malformations, gestational age and birth weight

Furthermore, there was an increased probability of receiving a disability pension among men diagnosed with hypospadias. However, after adjusting for the presence of other malformations, birth weight and gestational age in regression models, only the decreased probability of being eligible for upper secondary school (adjusted OR 0.87, 95% CI 0.78–0.98) and the increased risk of receiving disability pension (adjusted OR 1.39, 95% CI 1.20–1.61) remained. There were no significant differences in probabilities of pursuing an academic career or entering marriage.

### Subgroup analyses

The trend in distal hypospadias was the same as in the whole group (Table 3). In crude analyses the risk of disability pension was elevated, the association did however only remain borderline significant when adjusting for other malformations, and was just short of remaining significant when birth weight and gestational age was added to the model (adjusted OR 1.20, 95% CI 0.98–1.48). In the adjusted analyses, only a lower probability of being eligible for upper secondary school (adjusted OR 0.77, 95% CI 0.64–0.92) remained significantly associated with hypospadias.

**Table 3:** Logistic regression models for the associations between distal hypospadias and socioeconomic outcomes.

	Crude	Model 1 *	Model 2 **	Model 3 ***
	OR (95% CI)	OR (95% CI)	OR (95% CI)	OR (95% CI)
Entered marriage	1.03 (0.91–1.16)	1.05 (0.93–1.19)	1.01 (0.89–1.15)	1.03 (0.91–1.18)
Disability pension	1.72 (1.42–2.09)	1.28 (1.06–1.56)	1.60 (1.31–1.95)	1.20 (0.98–1.48)
Eligible for upper secondary†	0.71 (0.60–0.85)	0.73 (0.61–0.87)	0.75 (0.63–0.90)	0.77 (0.64–0.92)
**Highest level of education**				
Elementary school	1.08 (0.94–1.24)	1.05 (0.91–1.21)	1.06 (0.92–1.22)	1.03 (0.89–1.19)
Upper secondary school	1	1	1	1
College	1.03 (0.93–1.13)	1.04 (0.94–1.14)	1.03 (0.94–1.15)	1.04 (0.94–1.15)
**Highest level of income**				
Low income	0.97 (0.82–1.16)	0.96 (0.81–1.15)	0.97 (0.81–1.16)	0.96 (0.81–1.15)
Median income	1	1	1	1
High income	0.89 (0.80–0.98)	0.91 (0.82–1.01)	0.92 (0.82–1.02)	0.93 (0.84–1.05)

^†^ Men born 1983 and onward included

* Other malformations

** Gestational age and birth weight

*** Other malformations, gestational age and birth weight

Among men diagnosed with proximal hypospadias there was an increased probability of receiving a disability pension (Table 4). This risk was further decreased, but not completely so, in the adjusted analyses (adjusted OR 1.94, 95% CI 1.11–3.37).

**Table 4:** Logistic regression models for the associations between proximal hypospadias and socioeconomic outcomes.

	Crude	Model 1 *	Model 2 **	Model 3 ***
	OR (95% CI)	OR (95% CI)	OR (95% CI)	OR (95% CI)
Entered marriage	0.82 (0.46–1.46)	0.87 (0.48–1.55)	0.78 (0.40–1.52)	0.82 (0.42–1.60)
Disability pension	4.24 (2.59–6.93)	2.32 (1.37–3.91)	3.13 (1.85–5.31)	1.94 (1.11–3.37)
Eligible for upper secondary†	1.96 (0.91–4.20)	1.98 (0.92–4.28)	2.21 (1.00–4.77)	2.23 (1.03–4.82)
**Highest level of education**				
Elementary school	1.26 (0.79–2.01)	1.16 (0.72–1.86)	1.14 (0.69–1.86)	1.06 (0.65–1.75)
Upper secondary school	1	1	1	1
College	1.06 (0.71–1.60)	1.07 (0.71–1.61)	1.12 (0.73–1.72)	1.13 (0.74–1.75)
**Highest level of income**				
Low income	1.15 (0.71–1.86)	1.13 (0.69–1.83)	1.09 (0.67–1.76)	1.07 (0.65–1.76)
Median income	1	1	1	1
High income	0.72 (0.45–1.16)	0.80 (0.49–1.28)	0.87 (0.53–1.46)	0.93 (0.56–1.56)

^†^ Men born 1983 and onward included

* Other malformations

** Gestational age and birth weight

*** Other malformations, gestational age and birth weight

There were no significant differences in the likelihood of pursuing an academic career, being a high-income earner or getting married in subgroup analyses by different phenotypes.

When further analyzing the group of men with hypospadias diagnosis who had received disability pension– 4.7% of the total sample vs. 2.4% for non-cases– 46,2% of them had also received a diagnosis of autism spectrum disorder, ADHD, behavioral disorder or intellectual disability. The increased odds of receiving a disability pension seen in all analyses was somewhat decreased, but not fully so, by stratifying for the presence of psychiatric illness, in the group without psychiatric illness (adjusted OR 1.38, 95% CI 1.14–1.68) vs. the group with psychiatric illness (OR 1.81, 95% CI 1.25–2.62).

## Discussion

The main objective of this register-based study, conducted with a matched cohort design, was to evaluate subsequent psychosocial outcomes in adult men born with hypospadias. Our findings indicate that these men do not differ from nonaffected men regarding the majority of the investigated psychosocial outcomes. However, a subgroup of the population born after 1982 was less likely to be eligible for upper secondary school. In addition, men with hypospadias were at an increased risk of receiving a disability pension, regardless of the hypospadias phenotype.

Apart from the slightly lower probability of being eligible for high school among the men born after 1982, no difference in educational or income level was detected. It has previously been suggested that boys 6 to 10 years of age with hypospadias have significantly lower social competency and more behavioral problems when compared with norm data,[15] and that there is an increased risk for neuropsychiatric disorders among men with hypospadias.[11] These studies may describe factors that interfere with academic achievement. However, in our analyses including the whole cohort the final educational level does not differ between affected and unaffected men. These results are in line with the studies showing that boys with hypospadias do not have impaired school grades[9], and that men with hypospadias do not differ in educational or occupational class.[12–14]

Marital status was used as a proxy variable for the ability to engage in a close relationship, and there were no differences in the probability of entering marriage, which is also in accord with previous findings.[13, 16]

The men with hypospadias were as an aggregate, and when further sub grouped as proximal, at increased risk of receiving a disability pension. Unfortunately it is not possible to analyze the underlying cause for granting the disability pension due to limitations regarding the registers. It would be reasonable to think that this finding was due to comorbidities such as low birth weight, prematurity, associated malformations or psychiatric illness and not hypospadias per se. The finding did however remain significant regardless of adjustments for these covariates, indicating that it is not due those factors alone.

At present the criteria for disability pension in Sweden are that “1) you are between 30 and 64 years old, 2) you will probably never be able to work full time due to illness, injury or disability, 3) your work capacity is diminished by at least one fourth in all jobs on the labor market. All jobs on the labor market include jobs that are arranged for persons with disabilities, such as employment with salary grants. Depending on how much the work capacity is diminished, full, three-quarter or one-quarter sickness compensation may be paid.”[17] As previously stated, we find it unlikely that the finding regarding disability pension is due to hypospadias specifically, but rather to some comorbidity associated with hypospadias. Nevertheless, we cannot rule out that some men’s work capacity has been severely impaired due to repeated hypospadias surgeries. The most common reasons for temporary sick leave in Sweden are psychiatric illness and musculoskeletal diagnoses.[18] Hypospadias are associated with limb defects. Solely by adjusting for the effect of registered associated malformations we cannot eliminate the risk, but find it unlikely, that the finding is due to that men with hypospadias have more musculoskeletal problems than non-affected men. Temporary sick leave due to psychiatric diagnoses is highly associated with subsequent disability pensions[19] and there are reports of an increased risk for psychiatric comorbidity and a lower health-related quality of life among those affected by hypospadias. [11, 20] Our subanalyses stratified according to the presence of psychiatric disease indicate that the increased risk of a disability pension is not only due to psychiatric comorbidity. When interpreting this finding one must, however, keep in mind that there may be an even larger skewness in the presence of psychiatric diagnoses that we cannot detect since data on psychiatric disorders in this study were collected from the NPR. NPR has had nationwide coverage since 1973 but include outpatient data only from 2001 and onward. Many who suffer from psychiatric illness are only seen in outpatient clinics, and may therefore not have received a diagnosis in the NPR. Furthermore, the routine for diagnosing neuropsychiatric disorders has evolved during the study period and thus the elder patients may not have received any of the diagnoses we have stratified for. The finding regarding disability pensions remains, however, unexplained and alarming and needs further investigation in order to identify the underlying cause and, if possible, counteract this trend.

### Strengths and limitations

The study method enabled us to study a large cohort over a long period of time and has the benefit of using objective and prospectively collected data. By conducting a register-based study, we diminished the risk of a selection bias that may be introduced in the type of clinical studies previously conducted in the field. Our data are objective and prospectively collected. Conclusions from this study are limited by the fact that our cohort is still relatively young. Although we have performed an age-matched analysis, some differences may not yet be detectable. Conclusions may also be affected by the improvement of surgical techniques that has taken place throughout the years.

## Conclusion

This study shows that men born with hypospadias in Sweden do not differ significantly from unaffected men regarding educational level, income or marital status. They are, however, at a greater risk of receiving a disability pension. The cause of this finding is unknown and warrants further investigation. The results from this study may be useful when it comes to reassuring parents that their children, despite being born with hypospadias, will have similar outcomes to those of children without hypospadias.
